# The initial Mayo Clinic experience using high-frequency oscillatory ventilation for adult patients: a retrospective study

**DOI:** 10.1186/1471-227X-6-2

**Published:** 2006-02-07

**Authors:** Javier D Finkielman, Ognjen Gajic, J Christopher Farmer, Bekele Afessa, Rolf D Hubmayr

**Affiliations:** 1 Intensive Care Unit, Saint Alexius Medical Center, 900 East Broadway, Bismarck, ND, USA; 2 Division of Pulmonary and Critical Care Medicine, Department of Internal Medicine, Mayo Clinic College of Medicine, Rochester, MN, USA

## Abstract

**Background:**

High-frequency oscillatory ventilation (HFOV) was introduced in our institution in June 2003. Since then, there has been no protocol to guide the use of HFOV, and all decisions regarding ventilation strategies and settings of HFOV were made by the treating intensivist. The aim of this study is to report our first year of experience using HFOV.

**Methods:**

In this retrospective study, we reviewed all 14 adult patients, who were consecutively ventilated with HFOV in the intensive care units of a tertiary medical center, from June 2003 to July 2004.

**Results:**

The mean age of the patients was 56 years, 10 were males, and all were whites. The first day median APACHE II score and its predicted hospital mortality were 35 and 83%, respectively, and the median SOFA score was 11.5. Eleven patients had ARDS, two unilateral pneumonia with septic shock, and one pulmonary edema. Patients received conventional ventilation for a median of 1.8 days before HFOV. HFOV was used 16 times for a median of 3.2 days. Improvements in oxygenation parameters were observed after 24 hours of HFOV (mean PaO_2_/FIO_2 _increased from 82 to 107, *P *< 0.05; and the mean oxygenation index decreased from 42 to 29; *P *< 0.05). In two patients HFOV was discontinued, in one because of equipment failure and in another because of severe hypotension that was unresponsive to fluids. No change in mean arterial pressure, or vasopressor requirements was noted after the initiation of HFOV. Eight patients died (57 %, 95% CI: 33–79); life support was withdrawn in six and two suffered cardiac arrest.

**Conclusion:**

During our first year of experience, HFOV was used as a rescue therapy in very sick patients with refractory hypoxemia, and improvement in oxygenation was observed after 24 hours of this technique. HFOV is a reasonable alternative when a protective lung strategy could not be achieved on conventional ventilation.

## Background

High-frequency oscillatory ventilation (HFOV) is a mode of mechanical ventilation in which gas exchange is achieved by oscillatory swings of airway pressure around a constant mean airway pressure (usually higher than that applied during conventional ventilation (CV)), through the rapid (3–15 Hertz) delivery of subnormal tidal volumes [1,2]. A renewed interest in HFOV has emerged in recent years, because animal data support the concept of reduced lung injury using this technique when assessed by several surrogate physiologic endpoints (surfactant function, mediators of inflammation, gas exchange) [1,3]; and because lung protection can be provided by a ventilatory strategy that limits both pulmonary overdistension and collapse of alveolar units [4,5]. Based on the encouraging findings with HFOV in animal models, several trials were undertaken in neonatal and pediatric patients [6-11]. None of these trials have shown a significant mortality benefit, however a recent metaanalysis published by the Cochrane library in preterm infants suggested that there may be a small reduction in the rate of chronic lung disease associated with the elective use of HFOV versus CV. [12].

In contrast to neonatal and pediatric populations, the experience of HFOV in adults is considerably smaller. Several case series in adult patients reported that HFOV improved oxygenation, and suggested a better outcome when applied early [3,13-18]. Two randomized controlled trials have compared HFOV versus CV. In the study by Derdak et al., 30-day mortality was not statistically different in patients treated with HFOV compared to those treated with CV [19]. However, an early but not persistent improvement in oxygenation was seen in patients treated with HFOV, and no differences in adverse events or multiorgan failure were found between the two treatment groups. An important limitation to this study is that patients in the CV arm received tidal volumes that now are recognized to be injurious to the lung and associated with poor outcomes. In the recent study by Bollen et al., there were no differences in survival without supplemental oxygen or on ventilator, mortality, therapy failure, or crossover [20]. However, this study was stopped prematurely after including 61 patients (37 received HFOV and 24 CV) because of low inclusion rates. As a consequence this study only had power to detect major differences in outcome, also the follow-up was incomplete in 7 patients, and the baseline oxygenation index (OI) of patients randomized to HFOV was worst compared to the CV arm (25 vs. 18 respectively). Therefore, the role of HFOV in the management of adult patients with acute lung injury remains to be defined.

HFOV was introduced in our institution in June 2003. The aim of this study is to review our first year of experience using HFOV in adult patients.

## Methods

In this retrospective study, we reviewed the electronic medical records of all the adult patients who were consecutively ventilated with HFOV (SensorMedics 3100B, Yorba Linda, CA) at the intensive care units of Mayo Medical Center, Rochester, Minnesota, from June 2003 to July 2004. Mayo Medical Center includes two hospitals, Saint Mary's Hospital and Rochester Methodist Hospital, with a total of approximately 1,300 beds and 140 intensive care unit (ICU) beds. HFOV was introduced to Mayo Medical Center in June 2003. Since then, there has been no protocol to guide the use of HFOV and all decisions regarding ventilation strategies and settings of HFOV were made by the treating intensivist.

The Mayo Foundation Institutional Review Board approved the study, and a waiver of informed consent was granted. Patients who did not authorize their medical records to be reviewed for research were excluded.

The data collected included demographics, comorbidities, etiology of the respiratory failure, ventilator modality, ventilator settings and duration of mechanical ventilation, blood gases, medical/surgical interventions, complications, mean arterial pressure, and the number and dosage of vasopressors one hour before and three hour after the initiation of HFOV, dosage of Fentanyl and Lorazepam 12 hours before and after the beginning of HFOV, use of a paralytic agent, ICU and hospital length of stay, and outcome.

The Acute Physiology and Chronic Health Evaluation (APACHE) II scores and predicted mortality rates. [21] and Sequential Organ Failure Assessment (SOFA) scores. [22] for the first day in the ICU were calculated as described in the literature. Additionally, the APACHE II and SOFA scores were calculated for the 24-hour period before HFOV was begun. Multiple organ failure was defined as the presence of a SOFA score greater than or equal to 2 in two or more organs. ARDS was defined as in the American-European Consensus Conference. [23]. The OI was calculated as the mean airway pressure × FIO_2 _× 100/PaO_2_. Septic shock was defined according to the ACCP/SCCM consensus conference [24]. Pneumonia was defined as proposed by the Centers for Disease Control and Prevention [25]. The predicted body weight for male patients was calculated as 50 + 0.91 [height (in cm)-152.4], and for female patients as 45.5 + 0.91 [height (in cm)-152.4] [5]. High dose steroid use was defined as a daily dose equal or greater than the equivalent of 60 mg of prednisone.

StatView 5.0 computer software (SAS Institute Inc., Cary, NC) was used for statistical analyses. Descriptive data are summarized as mean (standard deviation), median (interquartile range) or percentages. Comparisons were made using Wilcoxon signed rank test. The 95% confidence interval (CI) was calculated when needed. A *P *value < 0.05 was considered statistically significant.

## Results

HFOV was used in 15 medical patients since its introduction in June 2003. This report includes only 14 patients because one did not give research authorization. The mean (SD) age of the patients was 56 (20) years, 10 were males, all were Caucasians, and all had indicated that they wished to receive cardiopulmonary resuscitation in case of cardiac arrest. Baseline and pre HFOV characteristics of the patients are summarized in Table 1. The APACHE II and SOFA scores, and the PaO_2_/FiO_2 _before the institution of HFOV did not differ significantly from the admission values (Table 1). Eleven patients had ARDS, nine due to pneumonia, one due to pneumonia and septic shock, and one due to pneumonia and severe sepsis. Two patients had unilateral pneumonia with septic shock, and one patient had pulmonary edema and hemorrhage due to acute myocardial infarction. Two patients received a recent bone marrow transplant as treatment for acute lymphocytic leukemia, and one was in blast crisis due to acute myeloid leukemia. None of the patients had chronic obstructive pulmonary disease but two had interstitial lung disease. All the patients had multiorgan failure when HFOV was begun (Table 1).

**Table 1:** Characteristics of the patients on admission to the ICU and pre-HFOV

	**First 24 hours**	**Pre-HFOV**
Predicted APACHE II hospital mortality (%)	83 (49–92)	
APACHE II score	35 (23–40)	35 (33–38)*
SOFA score	12 (8–18)	15 (10–16)†
PaO_2_/FIO_2_	80 (32)	73 (20)‡
Multiorgan failure (number of patients)	11	14

Data is presented as mean (SD) or median (25^th ^– 75^th ^percentiles).

* *P *= 0.18, † *P *= 0.07, ‡ *P*= 0.81, using Wilcoxon signed rank test.

All patients received CV for a median (IQR) of 1.8 (0.7–3.0) days before HFOV was started. Gas exchange parameters and ventilator settings on admission, immediately before the institution of HFOV and after cessation of HFOV are presented in Table 2. HFOV was used 16 times (in one patient HFOV was utilized three different times during his ICU stay) for a median (IQR) of 3.2 (0.9–6.5) days. Improvements in oxygenation parameters were observed after 24 hours of HFOV (Table 3). Detailed HFOV settings and gas exchange information are shown in Table 3. In one patient HFOV was discontinued because of severe hypotension that was unresponsive to fluids (patient is described in the next paragraph). One patient (the first of our series) who had unilateral pneumonia and septic shock was withdrawn from HFOV because of equipment failure, the high Δ P incapacitated the function of the gravity fed humidifier, which alarmed continuously. This problem was solved by placing a three way stopcock in the line coming from the water reservoir, and by pumping water manually with a syringe from the reservoir into the humidifier. No subsequent cases of equipment failure were noted in this series. In the 6 survivors, the median (IQR) length of time from the end of HFOV to being completely weaned from mechanical ventilation was 17.0 (12.0–21.0) days.

**Table 2:** Conventional ventilation settings and gas exchange before and after HFOV

	**Initial**	**Immediately Before HFOV**	**After HFOV**
Number of patients	14	14*	12
Volume control ventilation	12	9	7
Pressure control ventilation	2	7	6
Tidal volume (mL/Kg †)	7.1 (2.1)	6.1 (1.0)	6.2 (1.3)
PEEP (cm H_2_O)	13 (5)	14 (4)	13 (4)
Peak airway pressure (cm H_2_O)	37 (10)	39 (5)	34 (7)
Plateau pressure (cm H_2_O)	35 (11)	35 (6)	32 (11)
Mean airway pressure (cm H_2_O)	19 (6)	24 (4)	20 (5)
Respiratory rate (per minute)	24 (7)	28 (6)	24 (4)
Minute ventilation (L/minute)	12 (4)	11 (3)	10 (4)
FIO_2_	0.95 (0.11)	0.91 (0.16)	0.75 (0.23)
PaO_2 _(mm Hg)	81 (33)	63 (8)	67 (17)
PaCO_2 _(mm Hg)	52 (14)	57 (17)	56 (12)
pH	7.28 (0.12)	7.26 (0.15)	7.28 (0.15)
PaO_2_/FIO_2_	89 (47)	73 (20)	96 (34)
Oxygenation index	27 (16)	35 (10)	26 (16)

Data is presented as mean (SD).

* In one patient HFOV was used three times; † Predicted body weight;

**Table 3:** HFOV settings and gas exchange

	**Baseline**	**6 hours**	**24 hours**	**Last†**
N	16	12	11	16
Paw (cm H_2_O)	31 (3)	31 (3)	30 (2)	29 (4)*
Δ P (cm H_2_O)	64 (9)	64 (9)	64 (9)	70 (16)
Frequency (Hz)	5.3 (0.8)	5.3 (0.7)	5.3 (0.6)	5.2 (0.8)
FIO_2_	0.91 (0.14)	0.81 (0.14)*	0.73 (0.16)*	0.71 (0.21)*
PaO_2 _(mm Hg)	73 (29)	70 (14)	75 (9)	69 (11)
PaO_2_/FIO_2_	82 (30)	90 (27)*	107 (23)*	105 (33)*
Oxygenation index	42 (12)	37 (13)	29 (7)*	31 (13)*
PaCO_2 _(mm Hg)	51 (11)	49 (8)	48 (12)	50 (10)
pH	7.28 (0.16)	7.33 (0.12)	7.36 (0.11)	7.32 (0.16)

Data is presented as mean (SD).

* *P *< 0.05 when comparing with baseline values using Wilcoxon signed rank test. † Last settings and gas exchange before transitioning to conventional ventilation after a median (IQR) of 3.2 (0.9–6.5) days of HFOV.

Regarding patient's hemodynamics, no significant change in the mean (SD) arterial pressure was noted one hour before compared to three hours after the initiation of HFOV (73 (9) vs. 71 (7) mm Hg, *P *= 0.42). In one patient who was receiving multiple vasopressors when HFOV was started, this technique was discontinued since it was felt that the patient was too unstable for this mode of ventilation. Excluding this patient, neither the dosage (data not shown) nor the number of vasopressors changed when comparing one hour before to three hours after the initiation of HFOV (median number of vasopressors before and after HFOV were 1.0 (0.0–1.0) and 1.0 (0.0–1.0) respectively, *P *= 0.33).

During the admission to the ICU, all the patients had bronchoalveolar lavages performed (5 had a bronchoscopy done during HFOV), 9 were treated with nitric oxide, 9 patients received high dose steroids (three of them for unresolving ARDS), 7 had renal replacement therapy, and 5 had a tracheostomy placed. Two patients had pneumothorax before the institution of HFOV (no patient had a pneumothorax while on HFOV). No patient received prone ventilation. The mean (SD) dosing infusion for Fentanyl before and after the implementation of HFOV was 115 (54) vs. 144 (51) μg/hour (*P *= 0.052), and that of Lorazepam 3.7 (3.2) vs. 4.2 (2.3) mg/hour (*P *= 0.36). All but one patient received a paralytic agent; in nine, the paralytic agent was started to facilitate CV; in the other four the paralysis was needed after the beginning of HFOV to facilitate this technique.

Eight patients died (57 %, 95% CI: 33–79): life support was withdrawn in six patients, and two suffered cardiac arrest (in one of them while on HFOV). The median (IQR) ICU and hospital length of stay were 17.4 (7.7–23.9) and 27.4 (12.3–50.8) days respectively. Of the 6 survivors, all had a clinical diagnosis of critical illness polyneuropathy, 5 were discharged to a rehabilitation center and one to a nursing home. At the moment of writing this report (March 2005), all the survivors are still alive; this represents more than 12 months survival for four patients and more than 6 months survival for 2 patients.

## Discussion

This study describes the first 14 adult patients that received HFOV at our institution. The clinical characteristics of these patients (all had multiorgan failure, most had ARDS and most received nitric oxide, high dose steroids, and neuromuscular blocking agents), and the poor gas exchange prior to HFOV (PEEP 14 cm H_2_O, PaO_2_/FiO_2 _73, and OI 35) along with high plateau pressures despite a tidal volume of 6 mL/Kg of predicted body weight, suggest that HFOV was used as a rescue therapy for very sick patients with refractory hypoxemia in whom a protective lung strategy could not be achieved on CV. In fact, our cohort represents one of the sickest ever published (Table 4); yet 30-day mortality was similar to these reports (Table 4). Since three studies have shown that early initiation of HFOV was more likely to result in improved survival [3,15,18], we can hypothesize that the relative good outcome of our patients could be in part explained by the fact that they received CV for approximately only two days before HFOV compared to 3 to 7 days in previous series (Table 4). Additionally, a *post hoc *analysis of the recent randomized controlled trial comparing HFOV vs. CV suggested that HFOV had a better treatment effect compared to CV in patient with higher OI at baseline [20]. However, a good outcome comparison between our and previous studies can not be made because the later were done before there was a clear consensus that low plateau pressures and low tidal volumes were lung protective.

**Table 4:** Summary of all the observational studies and randomized control trials with HFOV in adult patients

						**Baseline**			
**Author**	**N**	**Period**	**CV (days)**	**Paralysis**	**APACHE II score**	**PaO**_2_**/FiO**_2_	**OI**	**Mortality (30 days) (%)**	**Mortality (hospital) (%)**
Mehta	156	1998–2002	5.6	90%	24 (1st day in ICU)*	91	31	62	
David	42	1998–2001	3.0†	NR	28 (baseline)†	94†	23†	43	52
Mehta	24	1997–1999	5.7	22/24	22 (baseline)	99	33	67	
Fort	17	NR	5.1	All	23 (baseline)	66	49	53	
Andersen	16	1997–2001	7.2	11/16	27 (baseline)	92	28		31 (3 month)
Cartotto	6	1999–2000	4.8	All	16	92	32		83
Claridge	5	1998	NR	All	29	52	NR		20

Derdak ‡	75	1997–2000	2.8	All	22 (baseline)	114	24	37	
	73		4.4	NR	22 (baseline)	111	27	52	

Bollen ‡	37	1997–2001	2.1	NR	21 (baseline)	96	25	43	
	24		1.5	NR	20 (baseline)	123	18	33	

Our cohort	14	2003–2004	1.7	13/14	36 (1st day in ICU)*	73	35	57	57

Unless specified, data are presented as means. CV: conventional ventilation prior HFOV; OI: oxygenation index; NR: not reported

* APACHE II before the institution of HFOV did not differ significantly from the first day in the ICU. † Median. ‡ Randomized controlled trials in adults. The upper and lower lines display the HFOV and CV arms respectively. In these two studies no significant differences among any of these variables were found, but for the OI in the study by Bollen et al.

Like previous studies [3,13,15,18,19], we observed improvement in oxygenation parameters after HFOV was started. In the study by Derdak, this improvement was attributed to the higher mean airway pressure used during HFOV compared to CV [19]. As expected, the mean airway pressure was increased when our patients were switched from CV to HFOV (from 24 to 31 cm of H_2_O; *P *= 0.0007). Additionally, with the application of a higher airway pressure during HFOV, a reduction in the cardiac output along with increases in the pulmonary arterial occlusion and central venous pressures have been reported [13,15,18,19]. But in none of these studies, these changes resulted in a clinical significant decrease of the mean arterial pressure. Similarly, we noted no change in mean arterial pressure or vasopressor requirement following the institution of HFOV.

It is important to acknowledge, that most of the patients in our cohort received several interventions with no clear outcome benefits to treat hypoxia in addition to HFOV. The majority of the patients received nitric oxide despite that neither alone. [26,27] nor in combination with HFOV [28] have been shown to reduce mortality in ARDS. Likewise, most of them received corticosteroids even though some controversial evidence suggests a beneficial effect in late non-resolving ARDS [29,30]. Additionally, two patients with unilateral lung injury were placed on HFOV, and even though this technique might prevent overdistension of the uninjured lung and allow recruitment on the injured lung, this use of HFOV has never been reported with the exemption of a case report in which independent lung ventilation with HFOV was employed for the management of asymmetric acute lung injury [31]. It is possible to speculate that these three interventions were used in these very sick patients because it has become increasingly clear that interventions are more efficacious in patients with higher risk of dying [32-34], however this practice most likely represents a rescue effort to save the life of these dying patients.

Most of our patients required a paralytic agent, even though in only a minority it was started to facilitate the management of HFOV exclusively. In previous studies using HFOV, most of the patients were paralyzed (Table 4). For example, in the study by Derdak the patients who were in the HFOV arm were paralyzed per protocol [19], and in the largest observational study up to date, 90% of 156 patients were paralyzed as well [18]. This requirement of neuromuscular blockade could be explained by the fact that by design, the 3100B has insufficient flow to meet adult patients' inspiratory demands, and when a respiratory effort produce a reduction of the airway pressure below 5 cm of H_2_O, the 3100B stops the oscillations, as this is interpreted as a circuit disconnection [15]. In contrast, no difference in the use of paralytic agents was noted when low versus traditional tidal volumes were compared [5]. Whether paralysis is a mandatory requirement of HFOV or just an unavoidable need of patients with severe refractory hypoxemia requiring mechanical ventilation is unclear. If the former proves to be true -since the current recommendation regarding the use of neuromuscular blocking agents in an ICU states that they should be used only when all other means have been tried without success [35]- this represents a clear disadvantage of HFOV over CV with low tidal volumes.

Our study has several limitations. The data were collected retrospectively, the sample size is small and represent a mixed population (three patients without ARDS), there is no control group, there was no standard protocol for the use of HFOV or for sedation, and the decision to use HFOV was made by the treating intensivists. Also, no trauma or burn patients were included in this series. Despite these limitations we found that HFOV is a viable rescue therapy for patients with severe refractory hypoxemia. At present, HFOV remains in the same category of other interventions that have been shown to improve oxygenation but not the survival rate of patients with ARDS (like nitric oxide. [26,27], prostaglandin E1. [36,37], and prone position [38]). HFOV has the theoretical advantages of providing lung protection by delivering subnormal tidal volumes and limiting both pulmonary overdistension and collapse of alveolar units

## Conclusion

During our first year of experience, HFOV was used as a rescue therapy in very sick patients with refractory hypoxemia. Improvement in oxygenation was observed after 24 hours of instituting this technique. HFOV is a reasonable alternative to CV when a protective lung strategy could not be achieved. This study, like previous reports, supports the need for a definitive randomized, control trial of HFOV vs. CV in adult patients with ARDS.

## List of abbreviations

APACHE: Acute Physiology and Chronic Health Evaluation

ARDS: Acute Respiratory Distress Syndrome

CV: Conventional ventilation

FIO_2_: Fraction Inspired of Oxygen

HFOV: High-Frequency Oscillatory Ventilation

ICU: Intensive Care Unit

IQR: Interquartile Range

OI: Oxygenation Index

PEEP: Positive End Expiratory Pressure

SD: Standard Deviation

SOFA: Sequential Organ Failure Assessment

## Competing interests

The author(s) declare that they have no competing interests.

## Authors' contributions

JDF: conception and design, acquisition, analysis and interpretation of the data, drafting the manuscript

OG: acquisition of the data, analysis and interpretation of the data, critical revision of the mauscript.

JCF: analysis and interpretation of data, critical revision of the manuscript.

BA: acquisition of the data, analysis and interpretation of the data, statistical analysis, critical revision of the mauscript.

RDH: conception and design, analysis and interpretation of the data, critical revision of the manuscript, supervision.

All authors read and approved the final manuscript.

## Pre-publication history

The pre-publication history for this paper can be accessed here:


